# Non-invasive ventilation for acute hypoxemic respiratory failure: intubation rate and risk factors

**DOI:** 10.1186/cc13103

**Published:** 2013-11-11

**Authors:** Arnaud W Thille, Damien Contou, Chiara Fragnoli, Ana Córdoba-Izquierdo, Florence Boissier, Christian Brun-Buisson

**Affiliations:** 1 Medical Intensive Care Unit, AP-HP, Henri Mondor University Hospital, Créteil, France; 2 Réanimation Médicale, CHU de Poitiers, 2 rue de la Milétrie, 86021, Poitiers, France; 3 INSERM U955, Créteil, France

## Abstract

**Introduction:**

We assessed rates and predictive factors of non-invasive ventilation (NIV) failure in patients admitted to the intensive care unit (ICU) for non-hypercapnic acute hypoxemic respiratory failure (AHRF).

**Methods:**

This is an observational cohort study using data prospectively collected over a three-year period in a medical ICU of a university hospital.

**Results:**

Among 113 patients receiving NIV for AHRF, 82 had acute respiratory distress syndrome (ARDS) and 31 had non-ARDS. Intubation rates significantly differed between ARDS and non-ARDS patients (61% versus 35%, *P* = 0.015) and according to clinical severity of ARDS: 31% in mild, 62% in moderate, and 84% in severe ARDS (*P* = 0.0016). In-ICU mortality rates were 13% in non-ARDS, and, respectively, 19%, 32% and 32% in mild, moderate and severe ARDS (*P* = 0.22). Among patients with moderate ARDS, NIV failure was lower among those having a PaO_2_/FiO_2_ >150 mmHg (45% vs. 74%, p = 0.04). NIV failure was associated with active cancer, shock, moderate/severe ARDS, lower Glasgow coma score and lower positive end-expiratory pressure level at NIV initiation. Among intubated patients, ICU mortality rate was 46% overall and did not differ according to the time to intubation.

**Conclusions:**

With intubation rates below 35% in non-ARDS and mild ARDS, NIV stands as the first-line approach; NIV may be attempted in ARDS patients with a PaO_2_/FiO_2_ > 150. By contrast, 84% of severe ARDS required intubation and NIV did not appear beneficial in this subset of patients. However, the time to intubation had no influence on mortality.

## Introduction

It is now well-demonstrated that non-invasive ventilation (NIV) can reduce intubation and mortality rates in patients with severe acute exacerbation of chronic obstructive pulmonary disease [1-3] or cardiogenic pulmonary edema [4]. By contrast, the beneficial effects of NIV remain unclear in patients with *de novo* acute hypoxemic respiratory failure (AHRF), that is, non-hypercapnic patients having acute respiratory failure in the absence of a cardiac origin or underlying chronic pulmonary disease. NIV is more likely to fail in hypoxemic patients [5], and NIV failure could be associated with increased mortality [6]. In unselected patients admitted to ICUs for AHRF, the rate of intubation is particularly high, reaching 60% [6,7], and their in-ICU mortality after intubation may exceed 60% [6-8]. Thus, NIV may improve outcome of patients who succeed in NIV by avoiding intubation, but may worsen outcome by delaying intubation in those having failed NIV.

Despite these concerns, surveys show that NIV is increasingly used in patients having AHRF and is initiated as first-line ventilatory support in 20% to 30% of such patients [5,7]. NIV has even been used as the first-line ventilatory support in patients having clinical criteria for acute respiratory syndrome (ARDS) [9,10] with a success rate of more than 50%, especially in patients with prompt improvement of oxygenation [9]. The increasing popularity of NIV in AHRF patients is supported by some studies showing that NIV markedly reduced intubation and mortality rates in immunosuppressed patients [11,12] or in selected surgical patients with AHRF [13,14].

However, few randomized controlled studies have been conducted in non-immunosuppressed patients with AHRF [15-18], and some of these included hypercapnic patients [15,16]. To date, only two randomized controlled studies have evaluated NIV in non-hypercapnic patients with AHRF [17,18], with one suggesting that NIV may reduce intubation rate and even mortality [18] and the other reporting no beneficial effects of continuous positive-end expiratory pressure without ventilatory assistance [17].

The aims of our study were to assess the rate of NIV failure in patients admitted for AHRF according to the presence and clinical severity of ARDS as recently defined [19], and to identify early predictors of NIV failure.

## Material and methods

This observational cohort study was conducted in our 24-bed medical ICU at Henri Mondor University Hospital in Créteil, France. The Institutional Review Board of the French Society for Respiratory Medicine approved this non-interventional study and waived the need for informed consent.

### Patients

All consecutive patients admitted during a three-year period (June 2008 to June 2011) and who received NIV as initial ventilatory support for AHRF were included. AHRF was defined as recent dyspnea with a respiratory rate >25 breaths/minute and/or sternocleidomastoid muscle activation with pulmonary infiltrates on chest X-ray, and a PaCO_2_ below or equal to 45 mmHg. We excluded patients who were intubated before ICU admission or intubated upon ICU admission without prior NIV, and patients for whom NIV was used with a “do not intubate” order. However, the outcome for those who were directly intubated for acute respiratory failure without prior NIV, and who met clinical criteria for moderate or severe ARDS was also collected. The study was conducted after the implementation of a nurse-driven NIV protocol which included prospective daily collection of clinical data and ventilatory parameters on a specific NIV monitoring form. When the NIV form was unavailable or incomplete, data were retrieved from the patient’s records.

### Non-invasive ventilation protocol and definitions

All stages of the protocol had been developed within a multidisciplinary working group including ICU physicians, nurses and respiratory therapists. The protocol aimed at empowering nurses to adjust the ventilatory settings and to improve the patient's tolerance to NIV following a simple decision algorithm. A daily NIV prescription by the physician indicated the duration of NIV sessions and targeted expiratory tidal volume (around 6 to 8 ml/kg) and oxygen saturation (SpO_2_) (≥94%).

Pressure-support (PS) ventilation was started using a pressure-support level of 8 cmH_2_O, a positive end-expiratory pressure (PEEP) level of 5 cmH_2_O, an inspiratory trigger of 3 L/minute, and a maximal inspiratory time of one second. The nurses then adjusted the ventilatory parameters, including pressure-support level and FiO_2_ (fraction of inspired oxygen), according to the protocol. Pressure-support level was gradually increased by 2 cmH_2_O steps to reach the target expiratory tidal volume and PEEP level was then adjusted as prescribed. FiO_2_ was gradually adjusted by 5% steps to reach the targeted SpO_2_. Non-invasive ventilation was applied intermittently for periods of at least two hours, with a minimal duration of six hours per day and was maintained until signs of respiratory distress improved. An algorithm was used by nurses in case of leaks, which involved first repositioning of the mask; second, reducing the PEEP level at 2 cmH_2_O; third, reducing the pressure-support level by steps of 2 cmH_2_O until the minimal expiratory volume was reached; and fourth, changing the mask interface.

A mobile cart containing all types and sizes of interfaces was available at the bedside during initiation of NIV. NIV was performed via a non-vented full-face mask (FreeMotion™ RT041, Fisher & Paykel, Auckland, New Zealand or Ultra Mirage™, Resmed, CA, USA), with an ICU ventilator using a dedicated NIV mode (Evita XL, Dräger, Lübeck, Germany, or Engström Carestation, GE Healthcare, Fairfield, CT, USA), equipped with a heated humidifier (MR850, Fisher & Paykel).

The following criteria were used for endotracheal intubation: loss of consciousness or psychomotor agitation hindering nursing care and requiring sedation; persistent hypotension (defined by systolic arterial blood pressure below 90 mmHg or mean arterial blood pressure below 65 mmHg) despite fluid resuscitation, or need for vasopressors; or two of the following criteria: frank worsening of respiratory distress under NIV, respiratory rate above 40 breaths per minute, SpO_2_ remaining below 90% despite FiO_2_ 100%, dependence to NIV for more than 12 hours, or pH <7.35. NIV failure was defined by the need for endotracheal intubation.

### Data collection

From the NIV monitoring forms, we analyzed the number and duration of NIV sessions, ventilator settings (pressure support level, positive end-expiratory pressure, FiO_2_), ventilatory parameters (SpO_2_, respiratory rate, expiratory tidal volume), hemodynamic parameters (heart rate, blood pressure and level of consciousness assessed using the Richmond Agitation-Sedation Scale (RASS) [20], with altered consciousness defined as a RASS <0. NIV tolerance and number of leaks were recorded on a 4-point scale, then dichotomized into “acceptable” (scored 2 to 3) or “poor” (scored 0 to 1) tolerance, and “minor” (scored 0 to 1) or “major” (scored 2 to 3) leaks, respectively. Blood gases were routinely measured one hour after initiation of NIV. Clinical data (respiratory rate, SpO_2_, blood pressure, heart rate, Glasgow coma score) and blood gases at admission before NIV initiation were retrospectively collected from the medical chart. We also recorded the occurrence of shock at admission or at initiation of NIV (defined by hypoperfusion signs and administration of at least 30 ml/kg fluids, dobutamine or vasopressors).

Patients were stratified according to the presence of clinical criteria for ARDS. The severity of ARDS was stratified using the recent Berlin definition [19], according to the value of oxygenation recorded within the first hour after NIV initiation, and classified as mild (201 ≤ PaO_2_/FiO_2_ (partial pressure of oxygen/fraction of inspired oxygen) ≤ 300 mmHg), moderate (101 ≤ PaO_2_/FiO_2_ ≤ 200 mmHg) or severe (PaO_2_/FiO_2_ ≤ 100 mmHg).

### Statistical analysis

Dichotomous variables are reported as number (percentage), and were compared using the chi-square or Fisher’s exact tests. Continuous variables are expressed as mean (± standard deviation) or as median and interquartile range (IQR, (25^th^ to 75^th^ percentiles)) after testing their normal distribution using the Shapiro-Wilk test. Groups were compared using the unpaired Student’s *t*-test or Wilcoxon rank-sum and Kruskall-Wallis tests, when appropriate. Odds ratios (OR) with 95% confidence intervals (CI) were used to describe differences between subgroups for NIV failure or death.

Survival without intubation was tested using Kaplan-Meier estimates and compared with the log-rank test. To evaluate independent factors associated with NIV failure, variables with a univariate *P*-value <0.10 were entered in a Cox proportional hazards model with time to intubation as the dependent variable, censoring data at ICU discharge. Among related variables, the most significant or clinically relevant was entered into the model in order to minimize the effect of colinearity. Because it was measured at 24 hours after admission, the general severity score SAPS 2 was not included in this analysis. Variables included in the model are reported with their corresponding hazard ratio (HR) and 95% CI. We considered two-tailed *P-*values <0.05 as significant. Statistical analyses were performed using the statistical software package STATA version 10.1 (Stata Corp., TX, USA).

## Results

### Patients

**Figure 1:**
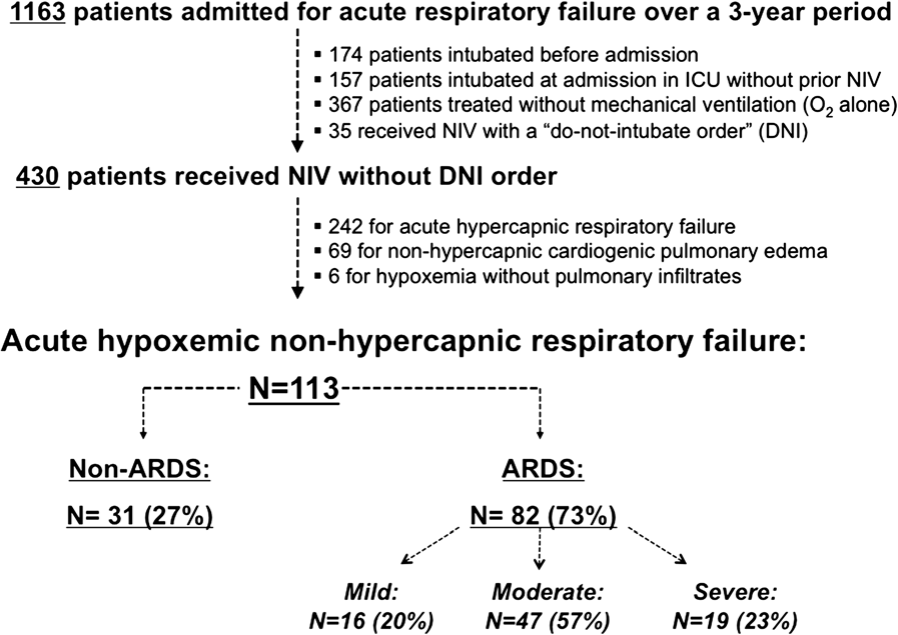
**Flow-chart of the study.** Among the 1,163 patients admitted for acute respiratory failure, 465 patients received NIV over a three-year period. After excluding 35 patients who received NIV with a “do not intubate” order, 430 received NIV of which 242 had acute hypercapnic respiratory failure and 188 had acute hypoxemic respiratory failure. After excluding 69 patients who received NIV for cardiogenic pulmonary edema and 6 patients without pulmonary infiltrates, 113 patients had non-hypercapnic acute hypoxemic respiratory failure. (ARDS, acute respiratory distress syndrome; NIV, Non-invasive ventilation).

Among 430 patients who received NIV during the study period, 188 had non-hypercapnic acute respiratory failure. After excluding patients with cardiogenic pulmonary edema and those without pulmonary infiltrates, 113 had *de novo* acute hypoxemic respiratory failure (Figure 1). Eighty-two patients had clinical criteria for ARDS at the time of NIV initiation, including 16 with mild (20%), 47 with moderate (57%) and 19 (23%) with severe ARDS. ARDS was due to bacterial pneumonia (n = 21), viral pneumonia (n = 7), pneumocystis jirovecii (n = 4), pneumonia without microbiological documentation (n = 24), aspiration (n = 5), alveolar hemorrhage (n = 6), drug induced pneumonia (n = 5), extra-pulmonary sepsis (n = 8), transfusion acute lung injury (n = 1), and fat embolism (n = 1). The 31 remaining patients without clinical criteria for ARDS (non-ARDS) had pneumonia (n = 17), atelectasis (n = 5), aspiration (n = 4), intra-alveolar hemorrhage (n = 2), pleural effusion (n = 2) or extra-pulmonary sepsis (n = 1). Overall, 50 patients (44%) were immunocompromised (Table 1), because of hematologic malignancy (n = 22), organ transplant (n = 10), HIV infection (n = 6), vasculitidis or steroid therapy (n = 4) or active/metastatic solid cancer (n = 8).

**Table 1:** Characteristics and outcomes of the patients receiving NIV for non-hypercapnic AHRF

	**No ARDS or mild ARDS (n = 47)**	**Moderate or severe ARDS (n = 66)**	***P***
Age, mean, years	60 (± 15)	61 (± 17)	0.72
Male sex, n (%)	30 (64%)	45 (68%)	0.62
SAPS II, points*	33 (23 to 39)	41 (30 to 51)	0.0014
Immunosuppression or cancer, n (%)	17 (36%)	33 (50%)	0.15
***Characteristics at admission before NIV***			
Sepsis, n (%)	27 (73%)	53 (70%)	0.72
Systolic arterial pressure, mmHg	131 (± 25)	127 (± 27)	0.43
Heart rate, beats/minute	114 (± 25)	110 (± 26)	0.43
Respiratory rate, cycles/minute	33 (± 7)	33 (± 7)	0.78
Glasgow coma scale, points*	15 (15 to 15)	15 (15 to 15)	0.75
pH, units*	7.4 (7.43 to 7.49)	7.45 (7.40 to 7.48)	0.24
PaCO_2_, mm Hg	34.6 (± 6.3)	35.4 (± 5.0)	0.43
PaO_2_, mm Hg	77 (± 41)	85 (± 54)	0.43
Bicarbonates, mmol/L	25.0 (± 4.8)	24.5 (± 5.0)	0.58
Lactates, mmol/L*	1.4 (1.0 to 2.0)	1.9 (1.1 to 2.6)	0.054
***At 1 h of NIV initiation***			
pH*	7.46 (7.39 to 7.49)	7.42 (7.38 to 7.46)	0.03
PCO_2_, mm Hg	36.1 (± 6.6)	37.8 (± 7.4)	0.21
PaO_2_/FiO_2_, mm Hg*	266 (219 to 330)	124 (94 to 164)	<0.0001
PEEP level, cm H_2_O	4.7 (± 1.1)	4.5 (± 1.1)	0.46
PS level, cm H_2_O*	8.0 (6.5 to 10)	8.0 (6 to 8)	0.46
Tidal volume, ml	579 (± 174)	613 (± 173)	0.37
Respiratory rate, breaths/minute*	34.5 (25 to 40)	32 (26 to 37.5)	0.45
***Outcome***			
Duration of NIV Day 1, hours*	6.8 [4.0 to 10.0]	5.2 [2.0 to 10.0]	0.19
Total duration of NIV, days*	2.0 [1.0 to 3.0]	1.0 [1.0 to 2.5]	0.17
Rate of NIV Failure, n (%)	16 (34%)	45 (68%)	0.0003
Length of stay in ICU, days*	8.0 [5.0 to 15.0]	10.0 [6.0 to 14.0]	0.53
ICU mortality, n (%)	7 (15%)	21 (32%)	0.04

Values are given as mean (± standard deviation) or as median [IQR, 25^th^ to 75^th^] for non-normally distributed variables (*).

AHRF, Acute Hypoxemic Respiratory Failure; ARDS, Acute Respiratory Distress Syndrome; ICU, Intensive Care Unit; NIV, Non-Invasive Ventilation; SAPS II, Simplified Acute Physiology Score II.

### Rates of NIV failure and in-ICU mortality

**Figure 2:**
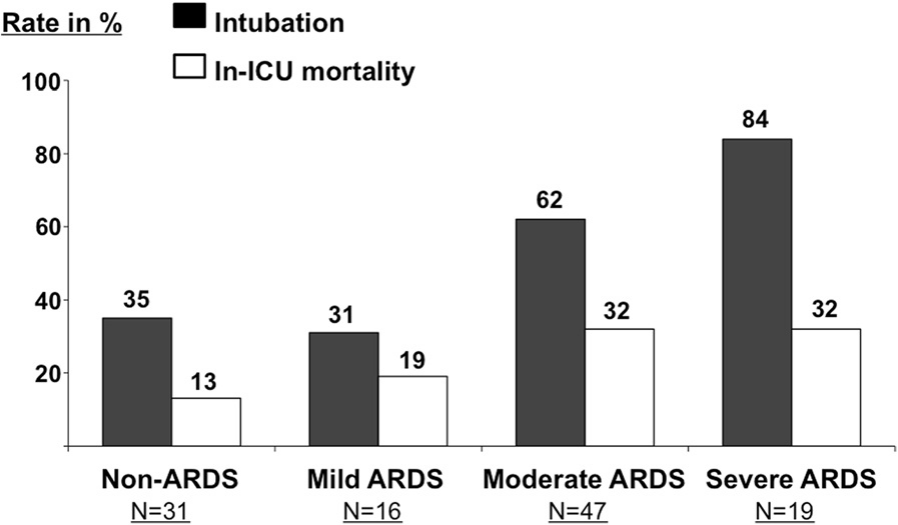
**Rates of NIV failure and in-ICU mortality (expressed in %) according to clinical criteria for acute respiratory distress syndrome (ARDS) and clinical severity of ARDS using the Berlin definition.** Intubation rate was significantly different between the four groups (*P* = 0.001) but not the mortality rate (*P* = 0.22). Intubation and mortality rates were higher in patients with moderate or severe ARDS than in patients with mild or without clinical criteria for ARDS.

**Figure 3:**
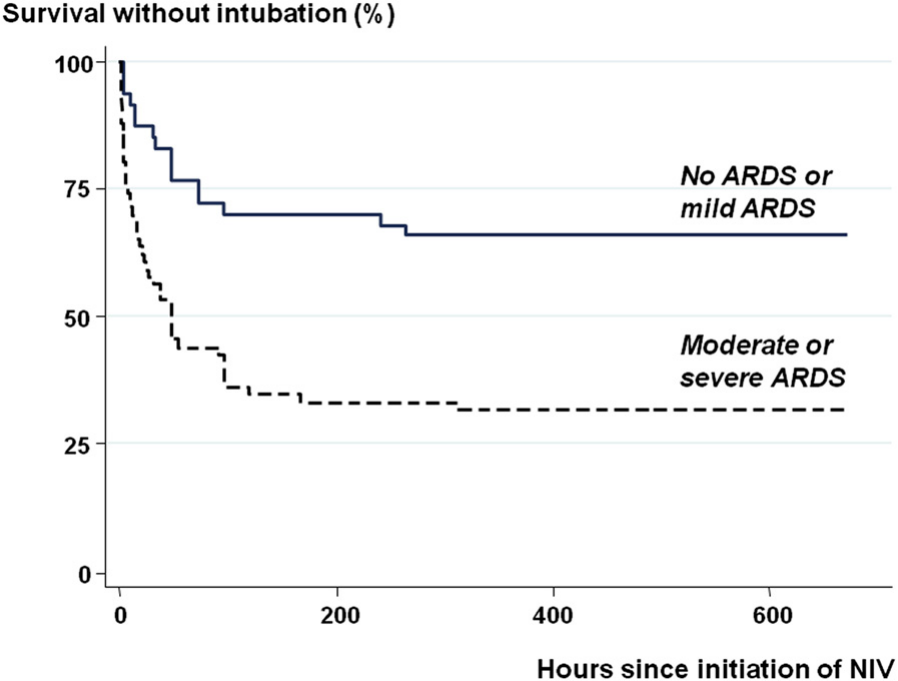
**Kaplan-Meier estimate of survival without intubation according to presence of ARDS and its severity at presentation, stratified as no ARDS or mild ARDS (solid line) or moderate or severe ARDS (dashed line).** The difference between the two groups was highly significant (*P* <0.0001, log-rank test). (ARDS, acute respiratory distress syndrome).

The rate of intubation was 61% (50/82) in ARDS and 35% (11/31) in non-ARDS patients (*P* = 0.015). This rate did not differ between patients without ARDS or those with mild ARDS (*P* = 0.71), but increased with increasing clinical severity of ARDS from 31% (5/16) in mild, 62% (29/47) in moderate, to 84% (16/19) in severe ARDS (*P* = 0.0016) (Figure 2). Patients with moderate or severe ARDS were twice as likely to fail NIV (45/66, 68%) than those with no ARDS or with mild ARDS (16/47, 34%); (OR = 4.15, 95% CI: 1.78 to 9.70; *P* = 0.0004) Survival analysis showed that intubation rates differed markedly (*P* <0.00001, Log-rank test) between patients with no or mild ARDS and those with moderate or severe ARDS (Figure 3).

Overall in-ICU mortality rate was 25% (28/113), and tended to be higher in patients with ARDS (24/82, 29%) than others (4/31, 13%, *P* = 0.07) (Figure 2). The mortality rate of patients with moderate or severe ARDS was also twice as high (21/66; 32%) as those with no or mild ARDS (7/47; 15%) (OR = 2.7; 95% CI: 1.003 to 7.09; *P* = 0.041).

**Figure 4:**
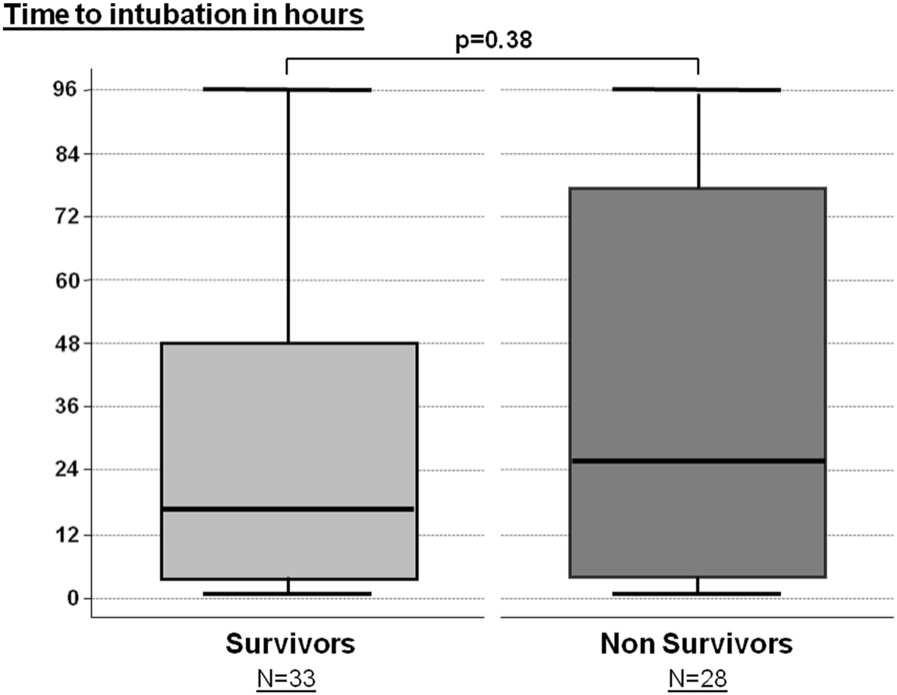
**Box-plots indicating the median delay (25**^**th **^**to 75**^**th **^**percentiles) between NIV initiation and intubation in patients intubated within the first 96 h.** The time to intubation was similar in survivors (at left) and non-survivors (at right). Only five patients (three survivors and two non-survivors) were intubated beyond 96 hours.

**Figure 5:**
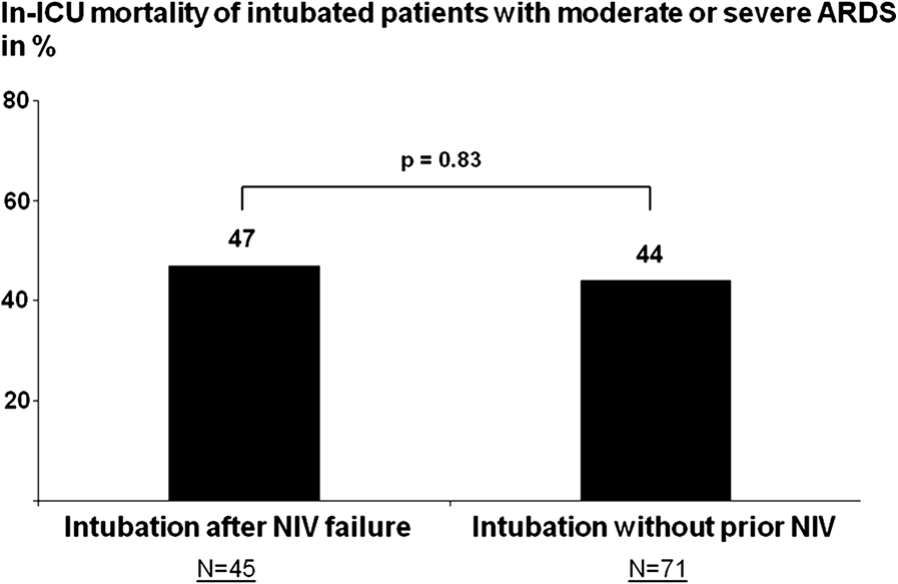
**Rate of in-ICU mortality in patients with moderate or severe ARDS.** No difference was found in patients who were intubated after NIV failure as compared to those who were directly intubated for acute respiratory failure without prior NIV (at right).

Among intubated patients, the overall in-ICU mortality rate was 46% (28/61). Thirty-three patients (54%) were intubated within the first 24 hours while the 28 patients remaining (46%) were intubated beyond 24 hours. The delay between NIV initiation and intubation had no influence on outcome with a similar time to intubation in survivors and non-survivors (Figure 4). Among patients with moderate or severe ARDS, in-ICU mortality was similar in patients who were intubated after failure of NIV as compared to patients who were directly intubated without prior NIV (Figure 5).

### Factors associated with NIV failure

Prospective data from NIV monitoring forms were available for 81% (91/113) of patients. Patients who were not intubated received NIV during a longer duration than those who were intubated (3.3 ± 2.8 days versus 2.0 ± 2.0 days, *P* = 0.006). Patients who failed NIV had lower PEEP levels and poorer tolerance to NIV than patients who succeeded NIV. Patients who failed NIV had more often active cancer, shock on admission and moderate/severe ARDS. They also had a higher SAPS II score, a lower Glasgow coma score, and a lower PaO_2_/FiO_2_ ratio (Table 2). Among patients with moderate ARDS, those with a PaO_2_/FiO_2_ ratio <150 were at significantly higher risk of intubation: 20/27 (74%) vs. 9/20 (45%); HR = 2.3 (95% CI, 1.04 to 5.06); *P* = 0.04. The rate of microbiological documentation was similar in patients who succeeded NIV as compared to those who failed NIV: 44% (23/52) in the success group versus 49% (30/61) in the failure group (*P* = 0.70).

**Table 2:** Predictors of endotracheal intubation in patients receiving NIV for non-hypercapnic AHRF

	**NIV Success N = 52**	**NIV Failure N = 61**	**Univariate HR [95% CI]; *P*-value**	**Cox regression aHR [95% CI]; *P*-value**
Age, years	58.0 (± 17.1)	62.6 (± 14.4)	1.47 [0.99 to 1.02]; *P* = 0.142	-
Male sex, n (%)	36 (69%)	39 (64%)	0.85 [0.51 to 1.44]; *P* = 0.551	-
SAPS II	31.4 (± 10.9)	46.3 (± 18.2)	**1.05 [1.03 to 1.06]**; *P* <0.001	Not included
Immunosuppression/cancer, n (%)	20 (38%)	30 (49%)	1.30 [0.79 to 2.16]; *P* = 0.169	-
Cancer, n (%)	0 (0%)	8 (13%)	**4.36 [2.01 to 9.44]**; *P* <0.001	**2.74 [1.22 to 6.15]; *P*** **= 0.014**
***At Admission before VNI***				
Sepsis, n (%)	37 (71%)	43 (70%)	0.97 [0.56 to 1.68]; *P* = 0.913	-
Glasgow coma score	14.9 (± 0.5)	14.6 (± 1.2)	**0.84 [0.69 to 1.03]**; *P* = 0.098	**0.77 [0.63 to 0.96]; *P*** **= 0.018**
Respiratory Rate, breaths/minute	32.7 (± 7.0)	33.3 (± 6.6)	1.01 [0.97 to 1.05]; *P* = 0.517	-
pH	7.44 (± 0.06)	7.44 (± 0.08)	2.16 [0.05 to 89.3]; *P* = 0.685	-
PaO_2_, mm Hg	78.7 (± 43.0)	84.1 (± 53.2)	1.00 [0.99 to 1.01]; *P* = 0.442	-
PaCO_2_, mm Hg	34.8 (± 5.6)	35.3 (± 6.0)	1.01 [0.97 to 1.06]; *P* = 0.669	-
Bicarbonates, mmol/L	24.4 (± 4.8)	25.5 (± 5.0)	1.01 [0.96 to 1.06]; *P* = 0.547	-
Lactates, mmol/L	2.0 (± 1.8)	1.9 (± 1.2)	1.00 [0.85 to 1.18]; *P* = 0.940	-
Heart rate, beats/minute	114 (± 27)	110 (± 25)	0.99 [0.98 to 1.01]; *P* = 0.467	-
Shock, n (%)	4 (8%)	14 (23%)	**2.08 [1.14 to 3.79]**; *P* = 0.017	**1.89 [1.01 to 3.53]; *P*** **= 0.047**
Systolic arterial pressure, mmHg	134 (± 27)	124 (± 24)	**0.99 [0.98 to 1.00]**; *P* = 0.055	Not included
***At NIV initiation***				
Altered consciousness, n (%)	3 (6%)	8 (13%)	1.71 [0.81 to 3.59]; *P* = 0.158	-
pH	7.43 (± 0.06)	7.41 (± 0.10)	0.12 [0.00 to 4.31]; *P* = 0.244	-
PCO_2_, mm Hg	36.6 (± 6.7)	37.5 (± 7.5)	1.02 [0.98 to 1.05]; *P* = 0.327	-
PaO_2_/FiO_2_, mm Hg	211 (± 86)	163 (± 92)	**0.99 [0.99 to 0.99]**; *P* = 0.003	Not included
Moderate or severe ARDS, n (%)	21 (40%)	45 (74%)	**2.79 [1.57 to 4.95]**; *P* < 0.001	**2.57 [1.33 to 4.75]; *P*** **= 0.003**
PaO_2_/FiO_2_ <150 mm Hg, n (%)	12 (23%)	37 (61%)	**2.97 [1.77 to 4.99]**; *P* < 0.001	Not included
PEEP level, cm H_2_O	4.8 (± 1.0)	4.4 (± 1.3)	**0.77 [0.62 to 0.94]**; *P* = 0.011	**0.71 [0.57 to 0.88]; 0.002**
PS level, cm H_2_O	8.1 (± 2.2)	8.0 (± 1.9)	0.98 [0.86 to 1.13]; *P* = 0.915	-
Tidal volume, ml	576 (± 144)	619 (± 196)	1.00 [0.99 to 1.00]; *P* = 0.157	-
Respiratory rate, breaths/minute	32.7 (± 12.9)	33.9 (± 9.0)	1.01 [0.98 to 1.03]; *P* = 0.601	-
Important leaks, n (%)	2/43 (5%)	3/48 (6%)	1.39 [0.43 to 4.48]; *P* = 0.720	-
Poor tolerance, n (%)	3/43 (7%)	9/48 (19%)	**2.08 [1.00 to 4.30]**; *P* = 0.049	1.97 [0.89 to 4.30]; *P* = 0.09

Values are given as mean (± standard deviation) or proportion (%).

AHRF, Acute Hypoxemic Respiratory Failure; ARDS, Acute Respiratory Distress Syndrome; CI, 95% Confidence Interval HR; Hazard Ratio (aHR, adjusted HR); NIV, Non-Invasive Ventilation; PEEP, Positive End-Expiratory Pressure; PS, Pressure Support; SAPS II, Simplified Acute Physiology Score; VT, Tidal Volume.

Cox regression analysis showed that the risk of intubation was significantly associated with active cancer, a lower Glasgow coma score, shock, moderate/severe ARDS and a lower PEEP level (Table 2).

## Discussion

In our study, the intubation rate was higher in ARDS patients (61%) than in non-ARDS patients (35%). However, the 31% intubation rate in mild ARDS was close to that of non-ARDS, whereas it significantly increased up to 62% in moderate ARDS and to 84% in severe ARDS. After adjustment, underlying active cancer, moderate or severe ARDS, shock, lower Glasgow Coma Score (GCS) and lower PEEP level at NIV initiation were predictors of intubation. After NIV initiation, the time to intubation in patients who failed NIV did not influence outcome.

### NIV failure rate in patients with acute hypoxemic respiratory failure

In patients receiving NIV for AHRF, we found an overall rate of intubation of 54%, which is substantially higher than the 25 to 35% rate reported in randomized controlled trials evaluating NIV in AHRF [17,18]. However, in these two studies nearly 20 to 30% of the patients received NIV for cardiogenic pulmonary edema. Moreover, patients enrolled in such randomized studies are selected and, consistent with our results, intubation rates up to 60% have been reported in a series of unselected patients with AHRF of non-cardiac origin [6-8].

In their analysis of 147 ARDS patients receiving NIV as first-line therapy, Antonelli *et al*. [9] reported an intubation rate of only 46%. In this study, a high SAPS 2 (> 34) and low PaO_2_/FiO_2_ ratio (≤ 175 mmHg) after NIV initiation were the two risk factors of NIV failure, with an intubation rate of 78% (25/32) when both risk factors were present [9]. Although our overall intubation rate was higher, the intubation rate of patients presenting with the combination of these two criteria was strictly similar (79%, 27/34) and their mortality rate was likewise similar. However, using the SAPS 2 is clinically impractical since this score is computed only after 24 hours of admission, therefore taking into account the potential complications of intubation in patients who failed NIV within the first 24 hours.

### Time to intubation and impact on outcome

It has been suggested that NIV failure in patients with AHRF is independently associated with poor outcome as compared to patients intubated without prior NIV [6]. Therefore, it is essential to assess intubation rates and the impact of NIV failure on outcome in different subsets of the population with AHRF. It was recently suggested at an international conference that NIV may be a first line treatment in mild ARDS [21]. Our study supports this contention, as the intubation rate in patients with mild ARDS did not differ from that recorded in non-ARDS patients. In our patients with moderate or severe ARDS, however, the intubation rate was much higher (68%). Nevertheless, the mortality rate of patients failing NIV did not differ according to the time to intubation, in contrast with previous studies of patients with community acquired pneumonia [22] or receiving NIV during the post-extubation period [23]. We were unable to identify a time beyond which maintaining NIV may worsen outcome, and we believe that intubation should be decided according to standard criteria regardless of NIV duration.

It could also be argued that in our study NIV was used in moderately ill patients, while more severe patients would have been intubated without prior NIV. However, the rate of severe ARDS (19/82, 23%) in our population is close to that reported in the recent Berlin definition [19]. Mortality rates observed in intubated patients with ARDS are usually higher in observational studies than in randomized controlled studies and can reach 45% in unselected cohorts of patients [24,25]. Thus, our mortality rate of 48% in ARDS patients who failed NIV and required intubation is in line with these cohort studies of intubated patients.

### Predictive factors for NIV failure

As previously reported [6], immunosuppression had no influence on the success or failure of NIV; however, all eight patients in the subgroup having active or metastatic cancer failed NIV, and this factor remained significantly associated with NIV failure after adjustment (Table 2); thus, using NIV in this subgroup should be carefully considered. Not surprisingly, a low GCS and, to a lesser extent, shock were associated with NIV failure. Although neither controlled studies [17,18] nor surveys [5,7] found that the occurrence of shock was a risk factor of intubation, two others studies found that shock was associated with NIV failure [8,22].

Several studies found that hypoxemia was independently associated with NIV failure [7-9,22]. Our results confirm that stratification of patients according to the clinical severity of ARDS using the recent Berlin definition was clearly associated with the risk of NIV failure, with a low risk in patients with mild ARDS, increasing to 84% in those who had a PaO_2_/FiO_2_ ≤100 mmHg at initiation of NIV. However, a cut-off of 150 mmHg (a value close to that reported by Antonelli *et al*. [9]) appeared to more accurately segregate patients who failed from those who succeeded NIV. Therefore, whereas almost all patients with severe ARDS are likely to fail NIV, some patients with “moderate” ARDS might still benefit from a NIV trial.

### Limitations

Our study was conducted in a single unit with a long-standing experience in the practice of NIV and, therefore, our results may not be applicable to other centers with less extensive experience. Experience and nurse-driven protocols may improve NIV tolerance, and we report a poor tolerance rate of only 13% after one hour of NIV. In line with previous studies [5], poor tolerance was associated with NIV failure in univariate analysis but not after adjustment for other variables associated with NIV failure. However, whereas rate of NIV failure could be significantly reduced for hypercapnic patients in experienced centers [26], our rate of intubation was not lower in this series than in surveys including less experienced centers [5,7]. Another limitation is the retrospective nature of the study. However, prospective data collection of ventilatory parameters under NIV was available for a vast majority of our patients and, because of the availability of computerized medical charts for all patients, all those receiving NIV for AHRF could be analyzed.

## Conclusion

The major implications of our results are to easily identify hypoxemic patients who may benefit from NIV. Intubation rates did not exceed 35% in non-ARDS and mild ARDS and NIV may thus be used as the first-line ventilatory support, as recently suggested [21]. By contrast, 84% of severe ARDS required intubation and NIV does not appear beneficial in this subset of patients; however, the time to intubation after NIV failure did not seem to influence outcome of patients. In patients with moderate ARDS, NIV may be worth attempting in those having a PaO_2_/FiO_2_ ratio >150 in the absence of hemodynamic instability or altered consciousness; further studies are needed to define the most appropriate use of NIV in these patients.

## Key messages

• Intubation rates significantly differed between ARDS and non-ARDS patients and according to clinical severity of ARDS: 31% in mild, 62% in moderate and 84% in severe ARDS.

• NIV may be used as the first-line ventilatory support in mild ARDS whereas it does not appear beneficial in severe ARDS.

• In patients with moderate ARDS, NIV may be worth attempting in those having a PaO2/FiO2 ratio >150.

• The time to intubation after NIV failure did not seem to influence outcome of patients.

• Active cancer, shock, moderate/severe ARDS, lower Glasgow coma score and lower positive end-expiratory pressure level at NIV initiation were predictors of NIV failure.

## Abbreviations

AHRF: Acute Hypoxemic Respiratory Failure; ARDS: Acute Respiratory Distress Syndrome; GCS: Glasgow Coma Scale; ICU: Intensive Care Unit; NIV: Non-Invasive Ventilation; PEEP: Positive End-Expiratory Pressure; RASS: Richmond Agitation-Sedation Scale; SAPS: Sequential Organ Failure Assessment.

## Competing interests

The authors declare that they have no competing interests.

## Authors’ contributions

AWT, CBB and DC were responsible for study concept and design. DC, CF, ACI and FB were responsible for acquisition of the data. AWT, DC, CF, ACI, FB and CBB were responsible for the analysis and interpretation of the data. AWT and CBB drafted the manuscript.: AWT, DC, CF, ACI, FB and CBB were responsible for critical revision of the manuscript for important intellectual content. CBB and AWT performed the statistical analysis. CBB and AWT supervised the study. All authors read and approved the final manuscript.
